# Multi-Strategy Improved Aquila Optimizer with Adaptive Exploration and Individual-Level Stagnation Control: A Bio-Inspired Hybrid Metaheuristic and Its Engineering Applications

**DOI:** 10.3390/biomimetics11070483

**Published:** 2026-07-10

**Authors:** Oluwatayomi Rereloluwa Adegboye, Huseyin Kusetogullari, Afi Kekeli Feda

**Affiliations:** 1 Department of Management Information Systems, University of Mediterranean Karpasia, Northern Cyprus, TR-10 Mersin, Lefkosa 99010, Turkey; 2 Department of Computer Science, Blekinge Institute of Technology, 37141 Karlskrona, Sweden; 3 Advanced Research Centre, European University of Lefke, Northern Cyprus, TR-10 Mersin, Lefke 99010, Turkey

**Keywords:** bio-inspired algorithms, optimization, dynamic adaptation, Aquila Optimizer, Animated Oat Optimization, anomaly prediction

## Abstract

Metaheuristic algorithms remain a widely used class of solvers for solving complex, non-convex optimization problems where gradient information is unavailable, yet two failure modes continue to limit their practical reach: premature convergence caused by inadequate exploration diversity in late iterations and population stagnation that persists even when individual agents are nominally assigned to the exploration phase. This paper proposes the Stagnation-Aware Aquila Optimizer (SAAO), a hybrid algorithm that addresses both failure modes by embedding three targeted mechanisms into the Aquila Optimizer (AO) framework: (i) an adaptive exploration probability that responds to global fitness-improvement history; (ii) individual-level stagnation counters that force exploration re-entry for any agent that fails to improve for more than 30 consecutive iterations, regardless of the global phase schedule; and (iii) a diversity-maintenance module that reinitializes completely stagnant agents via random sampling or opposition-based learning. The biological repertoire of search operators is simultaneously enriched by incorporating four physics-grounded operators from the Animated Oat Optimization (AOO) algorithm centroid-guided dispersal, elite-guided dispersal, hygroscopic rolling, and spring ejection, alongside the original AO operators, yielding six complementary update rules partitioned equally between exploration and exploitation. The SAAO was evaluated against nine state-of-the-art algorithms on the CEC2015 benchmark and CEC2022 under identical experimental settings. The SAAO achieved the best Friedman mean rank on both suites and delivered competitive or superior performance against the nine baselines, with Wilcoxon rank-sum tests confirming statistically significant advantages over most competitors. On three classical engineering design problems, the SAAO achieved competitive outcomes. In a real-world equipment anomaly prediction task, an SAAO-optimized ensemble classifier attained 98.23% accuracy, surpassing the compared baseline models. These results establish SAAO as a robust and computationally tractable optimizer for both benchmark and applied settings.

## 1. Introduction

Optimization underlies nearly every quantitative decision in engineering and the sciences. From structural design [1] to deep neural network training [2,3], the ability to locate near-optimal configurations in high-dimensional, non-convex landscapes determines product quality, operational safety, and resource efficiency. For problems whose objective functions lack analytic gradients or whose evaluations are so expensive that gradient approximation is impractical, population-based metaheuristic algorithms constitute an efficient, computationally feasible class of general-purpose solvers [4]. Decades of sustained research have produced a rich ecosystem of such algorithms, spanning swarm intelligence [5,6], evolutionary computation [7,8], physics-inspired methods [9,10], and biology-inspired methods [11,12,13], each offering distinct strategies for balancing exploration of the search space against exploitation of promising regions.

The metaheuristic literature is not without well-documented criticisms. Wolpert and Macready’s No Free Lunch theorem [14] establishes that no single algorithm is universally superior across all optimization problems, making it imperative that any new proposal be evaluated across diverse benchmark categories and validated with appropriate statistical tests. Another concern is benchmark overfitting, where algorithms are tuned to perform well on specific test suites but fail to generalize to real-world applications [15]. A more fundamental critique, articulated most prominently by Sörensen [16], targets the proliferation of metaphor-driven metaheuristics whose claimed novelty is often superficial: many newly proposed methods rebrand existing search mechanics in fresh biological or physical language without delivering genuine algorithmic innovation and are frequently validated only on benchmark suites whose structure happens to flatter the proposed operators. In direct response, the present work positions the SAAO not as a new metaphor but as a variant of an established optimizer (AO): it adds improvement mechanisms. This work explicitly addresses all these concerns: the SAAO uses no problem-specific parameter tuning; evaluation spans two independent benchmark suites (CEC2015 and CEC2022) covering unimodal, multimodal, hybrid, and composition function categories; statistical validity is established via both Wilcoxon rank-sum tests and Friedman mean-rank tests; and generalization is demonstrated on three engineering design problems and one industrial machine-learning application.

The Aquila Optimizer (AO), proposed by Abualigah et al. [17], is one of the most widely adopted nature-inspired algorithms in recent years and has inspired numerous application studies. The AO models four hunting behaviors of the Aquila bird: high soaring with vertical stoop (expanded exploration, X1), contour flight with short glide attack (narrowed exploration, X2), low flight with slow descent attack (expanded exploitation, X3), and walk and grab prey (narrowed exploitation, X4). The phase switch between exploration and exploitation is governed by a fixed temporal threshold at iteration *t* = 2*T*/3, and the internal branching within each phase is controlled by a single Bernoulli trial with probability. Despite its simplicity and competitive baseline performance, the AO carries two well-recognized structural weaknesses. First, once the algorithm enters the exploitation phase, no mechanism exists to redirect individual agents that have become trapped in local optima. Stagnation persists silently until the run terminates. Second, the fixed branch probability within each phase cannot respond to emergent search dynamics, such as a sudden loss of population diversity that calls for a temporary boost in exploration intensity.

Prior research has attempted to address these weaknesses through various enhancement strategies [17,18]. These strategies have been introduced to improve diversity. Hybridization with the Grey Wolf Optimizer [19], Whale Optimization Algorithm [20], and other algorithms has extended the AO’s behavioral repertoire. However, these fixes generally operate at the population level or rely on static augmentation, leaving the core gap unaddressed: the absence of individual-level stagnation monitoring that can trigger corrective action for specific agents without disturbing the rest of the population. Independently, the Animated Oat Optimization (AOO) algorithm, proposed by Wang et al. [21], introduced a novel physics-based search paradigm inspired by the hygroscopic seed dispersal mechanism of *Avena sterilis*. AOO’s operators, cubic-decay parameter scheduling, sinusoidal bound oscillation, hygroscopic rolling, and spring ejection provide search diversity through physically distinct displacement principles rather than ad hoc noise injection. The rolling mechanism produces individual-specific step sizes governed by seed mass, awn length, and eccentricity parameters, while the ejection mechanism simulates stored-energy projectile motion that is specifically designed to escape obstacles (local optima). AOO’s plant-inspired perspective complements the AO’s animal behavior perspective, suggesting that their combination could provide search strategies at multiple scales and with qualitatively different escape dynamics.

This research proposes the Stagnation-Aware Aquila Optimizer (SAAO), which fuses the structural simplicity of the AO with the physics-grounded diversity of AOO and adds three novel control mechanisms that operate at the individual level. The integration is not a loose concatenation of operators but a restructured decision architecture in which six update rules, three per phase, are selected uniformly at random, ensuring that no single operator dominates and that behavioral diversity is maintained throughout the optimization process. The three new control mechanisms respond to real-time stagnation signals at both the individual and population levels, making the SAAO a unique AO variant that provides per-agent stagnation awareness as a first-class architectural component. The primary contributions of this work are as follows:

• Adaptive Exploration Probability: A three-tier probability rule adjusts the population-wide exploration likelihood based on the count of iterations without global improvement, rising from a time-decaying baseline to fixed levels under moderate and severe stagnation.
• Individual-Level Stagnation Control: Each agent maintains its own non-improvement counter; agents that exceed a threshold of 30 consecutive non-improving iterations are immediately redirected to exploration, regardless of the global phase clock, enabling targeted recovery without disrupting well-performing agents.
• Diversity Maintenance via Opposition-Based Reinitialization: Agents stagnant for more than 100 iterations are reinitialized using either uniform random sampling or three types of opposition-based learning (OBL), preserving population coverage and preventing premature convergence.

The remainder of this paper is organized as follows: Section 2 reviews related work. Section 3 presents the mathematical formulation of the AO, the AOO building blocks, and the complete SAAO algorithm. Section 4 reports experimental results, statistical analysis, behavioral characterization, engineering applications, and a real-world case study. Section 5 concludes with directions for future research.

## 2. Related Work

Since its proposal in 2021, the AO has inspired a substantial body of enhancement research organized around three broad strategies: introducing diversity-preserving mechanisms to escape local optima, restructuring the AO’s operators for improved scalability or simplicity, and hybridizing the AO with complementary algorithms.

### 2.1. Diversity and Local-Optima Escape

A persistent observation in the AO literature is that the base algorithm can stagnate in local optima as problem complexity scales. Gao et al. [22] addressed this by introducing a search control factor that dynamically modulates the exploration–exploitation balance, supplemented by mutation operators that perturb stagnant positions; the combined mechanism improved benchmark performance on tested functions. Varshney et al. [23] proposed two dynamic mechanisms: a dynamic random walk that continuously adapts agent step sizes to the current search state and a dynamic opposition-based learning scheme that generates candidates in the complementary search region, achieving improvements on constrained real-world benchmarks. Abualigah et al. [24] improved the base algorithm by adding opposition-based initialization and a mutation search strategy, reporting competitive results on both global optimization benchmarks and data clustering problems. Verma et al. [25] embedded chaotic map sequences into the AO’s candidate-generation mechanism alongside a single-stage evolutionary refinement step, confirming that chaotic initialization improves population diversity on the examined benchmark functions. Yu et al. [26] combined opposition-based candidate generation with Chaotic local search and a Restart strategy, achieving stronger performance on constrained engineering problems.

### 2.2. Structural Improvements

Zhao et al. [27] adopted the opposite direction, proposing a Simplified Aquila Optimization Algorithm that reduces the AO’s four-operator update structure to a more compact rule set while retaining competitive benchmark performance, demonstrating that a subset of the AO’s original complexity suffices for complex problems. Kan et al. [28] addressed the AO’s weakness on high-dimensional problems through a multi-strategy framework combining multiple complementary search behaviors, improving scalability on engineering benchmarks. Guo and Yi [29] compiled multiple incremental improvements into an enhanced AO variant validated across a range of application domains.

### 2.3. Hybridizations

The diversity of available metaheuristic algorithms has motivated extensive hybridization with the AO as the primary framework. Wang et al. [30] interleaved the AO’s high-soaring exploration with the phased dive-and-attack exploitation of the Harris Hawks Optimizer (HHO) via a time-varying selection probability, consistently outperforming compared optimizers on optimization benchmarks. Xiao et al. [31] broadened this in IHAOAVOA by fusing the AO with the African Vultures Optimization Algorithm, adding foraging and siege behaviors to the AO’s four hunting modes [31]. Bousmaha [32] combined the AO with the Whale Optimization Algorithm for feature selection, leveraging the WOA’s spiral position update to reinforce late-phase exploitation. Chu et al. [33] developed a hybrid AO–Sine Cosine Algorithm that uses the SCA’s update rules to diversify the AO’s search trajectory on numerical optimization problems. Akyol [34] hybridized the AO with the Tangent Search Algorithm, exploiting the TSA’s tangent-function perturbation as an escape mechanism for trapped agents. Chen et al. [35] fused the AO with the Pigeon-Inspired Optimization Algorithm, using PIO’s operators to complement the AO’s soaring model for PID parameter optimization.

Across all algorithms surveyed above, phase transitions and operator selection are governed by global signals, elapsed time, population rank, or aggregate diversity statistics that are applied uniformly to all agents. No existing AO variant and no comparison algorithm in this study maintains per-agent improvement history and uses it to individually redirect stagnant agents while leaving productive agents undisturbed. The SAAO fills this gap through individual-level stagnation counters that incur only *O*(*N*) bookkeeping per iteration, preserving the standard asymptotic complexity of the original AO.

## 3. Methodology

### 3.1. Aquila Optimizer

The Aquila Optimizer (AO) [32] is a population-based algorithm that models four hunting behaviors of the Aquila eagle. Given an objective function $f:{\mathbb{R}}^{D}\to \mathbb{R}$, bounds ${\left[LB,UB\right]}^{D}$, population size *N*, and maximum iterations *T*, the AO initializes a population matrix as defined in Equation (1).

(1) $$X={\left[X_{1},X_{2},\ldots ,X_{N}\right]}^{T}\in {\mathbb{R}}^{N\times D}$$

where each candidate solution is drawn uniformly according to Equation (2)

(2) $$X_{i,j}=rand\cdot \left(UB_{j}-LB_{j}\right)+LB_{j},\;i=1,\ldots ,N,\;j=1,\ldots ,D$$

The global best solution at iteration t is denoted $X_{\mathrm{best}\;}\left(t\right)$, and the population mean is given in Equation (3). Here, *t* denotes the current iteration counter, and *T* denotes the maximum (total) number of iterations of the run; the normalized ratio of both variables therefore grows monotonically from near 0 at initialization to 1 at termination and is used throughout both AOs as a time variable that drives the exploration-to-exploitation transition and the decay of several control factors.

(3) $$X_{M}(t)=\frac{1}{N}\sum_{i=1}^{N}\;X_{i}(t)$$

The AO enters the exploration phase when $t\leq \frac{2}{3}T$ and the exploitation phase otherwise. Within each phase, a single Bernoulli trial (rand ≤ 0.5) selects one of two update rules. The expanded exploration rule (X1) models high soaring according to Equation (4)

(4) $$X_{1}\left(t+1\right)=X_{\mathrm{best}\;}\left(t\right)\left(1-\frac{t}{T}\right)+X_{M}\left(t\right)-X_{\mathrm{best}\;}\left(t\right)\cdot \;\mathrm{rand}$$

The narrowed exploration rule (X2) models contour flight with Lévy-driven search as seen in Equation (5)

(5) $$X_{2}\left(t+1\right)=X_{\mathrm{best}\;}\left(t\right)\cdot \mathrm{Levy}\left(D\right)+X_{R}\left(t\right)+\left(y-x\right)\cdot \mathrm{rand}$$

where *X_R_* is a randomly selected population member, *t* denotes the current iteration, $y=r\cdot \cos\left(\theta \right)$ and $x=r\cdot \;\sin\left(\theta \right)$ define spiral coordinates with *r* as defined in Equations (6)–(8), and the Lévy step is given in Equation (9)

(6) $$r=r_{1}+U\times D_{1}$$

(7) $$\theta =-\omega \times D_{1}+\theta_{1}$$

(8) $$\theta_{1}=\frac{3\times \pi }{2}$$

The parameter *r*_1_ is an integer ranging from 1 to 20 and determines the total number of search cycles. Two small, fixed constants are also defined: *U* = 0.00565 and *ω* = 0.005. The variable $D_{1}$ takes an integer value from 1 up to the dimensionality of the search space (*Dim*). Together, these settings guide the AO algorithm to explore the solution space along a spiral trajectory. The narrowed-exploration rule X2 (Equation (5)) models the eagle’s contour flight, in which the bird flies low over the terrain following its shape before a short gliding attack. It combines three components. First, the term *X_best_*(*t*) *Levy*(*D*) anchors the search to the current best solution while injecting heavy-tailed Lévy steps that allow for occasional long jumps away from it. Second, the additive term *X_R_*(*t*), a randomly chosen population member, introduces population-driven diversity. Third, the spiral displacement (*y* − *x*)·*rand*, with $y=r\cdot \cos\left(\theta \right)$ and $x=r\cdot \;\sin\left(\theta \right)$ in Equations (6)–(8), sweeps the candidate around a contracting spiral so that the region surrounding the best solution is scanned at progressively finer radii. The net effect is a more focused narrowed exploration than the high-soaring rule X1: the agent still searches broadly via the Lévy and random-member terms, but its sampling is organized around a spiral path centered on promising areas.

(9) $$\mathrm{Levy}\left(D\right)=s\cdot \frac{u\cdot \sigma }{|v{|}^{1/\beta }},\;\sigma ={\left(\frac{\Gamma \left(1+\beta \right)\sin\left(\pi \beta /2\right)}{\Gamma \left(\frac{1+\beta }{2}\right)\beta \cdot 2^{\left(\beta -1\right)/2}}\right)}^{1/\beta }$$

Here, β=1.5,s=0.01, and $u,v∼N\left(0,\;1\right)$. Lévy flight foraging refers to a heavy-tailed random-walk search pattern observed in many foraging animals, in which a large number of short, local steps are interspersed with occasional very long jumps. Algorithmically, the step length is drawn from a Lévy stable distribution (here with stability exponent β = 1.5), so the term Lévy(D) in Equation (9) produces mostly small displacements that refine the current trajectory but, at random intervals, generates large excursions that relocate an agent to a distant, previously unexplored region. This property is what lets the narrowed-exploration rule X2 escape local basins: the bulk of the steps keep the contour-flight search coherent, while the rare long jumps provide the global reach needed to avoid premature convergence. The scale factor *s* and the standard-normal variables *u* and *v* calibrate the magnitude of these steps. The expanded exploitation rule (X3) models low flight according to Equation (10):

(10) $$X_{3}\left(t+1\right)=\left(X_{\mathrm{best}\;}\left(t\right)-X_{M}\left(t\right)\right)\times \alpha -\;\mathrm{rand}\;+\left(\left(UB-LB\right)\times \;\mathrm{rand}\;+LB\right)\times \delta$$

where α=δ=0.1 are small exploitation constants. The narrowed exploitation rule (X4) models walk-and-grab, governed by the quality function in Equations (11)–(13). The three coefficients appearing in Equation (11) are defined as follows and are used in X4 in this order: QF(t) is the quality function (Equation (12)), a slowly varying exploitation weight that scales the contribution of the best solution; $G_{1}=2\mathrm{rand}-1$ is a balance coefficient and controls the magnitude of the Lévy-driven movement; and $G_{2}=2\left(1-\frac{t}{T}\right)$ is a decreasing coefficient that falls linearly from 2 to 0 over the run and steers the step size of the agent toward the best solution. The notation “2*·rand*” (in QF and in G1) denotes uniform random numbers drawn in [0, 1] and then multiplied by 2. Each occurrence of *rand* is an independent draw, but within one expression 2*·rand* is one draw scaled by two.

(11) $$X_{4}\left(t+1\right)=QF\times X_{\mathrm{best}\;}\left(t\right)-\left(G_{1}\times X\left(t\right)\times \;\mathrm{rand}\;\right)-G_{2}\times \;\mathrm{Levy}\;\left(D\right)+\;\mathrm{rand}\times G_{1}$$

(12) $$QF\left(t\right)=t^{\frac{2\times \mathrm{rand}-1}{{\left(1-T\right)}^{2}}}$$

(13) $$G_{1}=2\times \;\mathrm{rand}-1,\;G_{2}=2\left(1-\frac{t}{T}\right)$$

All candidate positions are rebounded into ${\left[LB,UB\right]}^{D}$ via element-wise clipping, and greedy selection retains any position that improves fitness.

### 3.2. Animated Oat Optimization Algorithm

The Animated Oat Optimization (AOO) algorithm, proposed by Wang et al. [21], is a physics-grounded metaheuristic inspired by the hygroscopic seed dispersal mechanism of *Avena sterilis* (Animated Oat). Unlike animal-behavior-based algorithms that model predator–prey dynamics or swarm intelligence, AOO derives its search operators from the biomechanical processes governing plant seed dispersal, specifically the moisture-driven awn movements and energy-storage ejection mechanisms that enable Animated Oat seeds to navigate complex terrain and locate favorable germination sites. The Animated Oat is an herbaceous annual plant whose seeds exhibit a unique self-propelled dispersal strategy. After detaching from the parent plant, seeds disperse through three complementary mechanisms: (i) passive transport via wind, water, or animal carriers; (ii) hygroscopic rolling induced by cyclic humidity changes that cause the seed’s main awn to bend, coil, and straighten; and (iii) spring-like ejection when the awn encounters obstacles and releases stored elastic energy. These three biological imperatives—adaptive exploration, energy-efficient exploitation, and resilience to obstacles—motivate the corresponding algorithmic operators in AOO. For the present biomimetics-oriented contribution, it is important to state which of these biological principles are functionally transferred into the proposed SAAO and which remain computational abstractions. The functionally transferred principle is the coupling of search behavior to an agent’s internal state: just as a seed’s dispersal mode (passive transport, hygroscopic rolling, or spring ejection) is governed by its own physical condition and immediate surroundings, each SAAO agent’s update is conditioned on its own stagnation history rather than on a single global clock. The heterogeneous per-agent physics parameters (mass, awn length, and eccentricity) are likewise a genuine transfer, producing individual-specific step sizes analogous to morphological variation among real seeds. By contrast, the spiral coordinates, the Lévy jumps, the sinusoidal bound oscillation, and the projectile-motion formulae are computational abstractions: they reproduce the qualitative effect of the biological process (broad scanning, occasional long relocation, and obstacle escape) without claiming quantitative biological fidelity. Making this separation explicit ensures that the biomimetic contribution rests on the transferred state-dependent dispersal principle rather than on metaphorical labelling of the operators. AOO initializes a population of *N* candidate solutions in a *D*-dimensional search space bounded by ${\left[LB,UB\right]}^{D}$. The population matrix is defined as in Equation (14):

(14) $$X={\left[x_{1},x_{2},\ldots ,x_{N}\right]}^{⊤}\in {\mathbb{R}}^{N\times D}$$

where each candidate solution $x_{i}$ is initialized uniformly at random according to Equation (15)

(15) $$x_{i,j}=r\cdot \left({\mathrm{UB}}_{j}-{\mathrm{LB}}_{j}\right)+{\mathrm{LB}}_{j},\;i=1,2,\ldots ,N,\;j=1,2,\ldots ,D$$

where $r\in \left[0,\;1\right]$ is a uniformly distributed random number, and ${\mathrm{UB}}_{j}$ and ${\mathrm{LB}}_{j}$ denote the upper and lower bounds of the *j*-th dimension, respectively. A distinguishing feature of AOO is the assignment of individual-specific physics parameters to each agent, creating heterogeneous search behavior across the population. These parameters model the physical properties of Animated Oat seeds and are given in Equation (16)

(16) $$\begin{array}{c} m=0.5\cdot r/D\\ L=N\cdot r/D\\ e=0.5\cdot r/D\\ c={\left(1-\frac{t}{T}\right)}^{3} \end{array}$$

where *m* represents the mass of the seed, *L* is the length of the main awn, *e* is the eccentric rotation coefficient during rolling, *t* is the current iteration, and *T* is the maximum number of iterations. The cubic decay factor *c* governs the transition from exploration to exploitation, decreasing more apidly than linear decay in early iterations while preserving moderate values in the middle phase schedule that maintains exploration diversity longer than standard linear annealing. The exploration phase models the stochastic dispersal of seeds via external agents (wind, water, and animals), which naturally exhibit high randomness and enable global search across the solution space. The perturbation vector *W* is computed as provided in Equation (17)

(17) $$W=\frac{c}{\pi }\left(2r_{\dim}-1\right)⊗\mathrm{UB}$$

where $r_{\dim\;}$ is a *D*-dimensional vector of uniform random numbers in $\left[0,\;1\right]$, and ⊗ denotes elementwise multiplication. The factor c/π normalizes the perturbation magnitude, which decays over iterations as *c* decreases. Position updates follow a three-role assignment based on agent index as expressed in Equation (18)

(18) $$X_{t+1}(i)=\left\{\begin{array}{l} \frac{1}{N}\sum_{K=1}^{N}X_{T}(K)+W,\;\mathrm{if}\;\mathrm{mod}(i,N/10)=0\;[\text{Centroid-guided}]\\ X_{\mathrm{best}}+W,\;\;\;\;\;\mathrm{if}\;\mathrm{mod}(i,N/10)=1\;[\text{Elite-guided}]\\ X_{t}(i)+W,\;\;\;\;\mathrm{otherwise}\;\;\;\;\;\;[\text{Self-guided}] \end{array}\right\}$$

This three-role structure ensures behavioral diversity: centroid-guided agents explore around the population mean, elite-guided agents intensify search near the best-known solution, and self-guided agents perform independent local exploration. When no obstacles impede dispersal, seed position changes are achieved through hygroscopic rolling-a biomechanical process driven by moisture-induced stress gradients in the awn’s cellulose microfibrils. The rolling mechanism is modeled using eccentric rotation with physics-based torque formulas. First, an oscillating bound A is computed in Equation (19)

(19) $$A=\mathrm{UB}-\left|\frac{\mathrm{UB}\cdot t\cdot \sin\left(2\pi r\right)}{T}\right|$$

The sinusoidal term causes *A* to oscillate over iterations, creating varying search radii that prevent monotonic convergence and facilitate escape from local optima. The rolling displacement *R* incorporates the individual-specific physics parameters in Equation (20)

(20) $$R=\frac{m\cdot e+L^{2}}{D}r_{\dim}\left(-A,A\right)$$

where $r_{\dim\;}\left(-A,A\right)$ generates a D-dimensional random vector with values uniformly distributed in $\left[-A,A\right]$. The term ($m\cdot e+L^{2}$) produces heterogeneous step sizes across agents, as each possesses unique mass, eccentricity, and awn length values. AOO employs Lévy flights to add occasional large jumps that prevent premature convergence, computed in Equations (21) and (22)

(21) $$\mathrm{Levy}\left(D\right)=0.01\times \frac{\mu \cdot \sigma }{|\nu {|}^{1/\beta }}$$

(22) $$\sigma ={\left[\frac{\Gamma \left(1+\beta \right)\cdot \sin\left(\frac{\pi \beta }{2}\right)}{\Gamma \left(\frac{1+\beta }{2}\right)\cdot \beta \cdot 2^{\left(\beta -1\right)/2}}\right]}^{1/\beta }$$

where β=1.5 is the Lévy exponent, μ and ν are independent standard normal random vectors, and Γ denotes the gamma function. The Lévy distribution produces heavy-tailed step sizes, enabling rare but substantial jumps that explore distant regions. The final position update for rolling combines the displacement *R* with Lévy-weighted perturbation as expressed in Equation (23)

(23) $$X_{t}\left(i\right)=X_{\mathrm{best}\;}+R+c\cdot L\;é\mathrm{vy}\;\left(D\right)⊗X_{\mathrm{best}\;}$$

When seeds encounter obstacles during dispersal, the main awn stores elastic energy and subsequently releases it through a spring-like ejection. This mechanism is modeled as projectile motion, providing an escape pathway from local optima analogous to obstacle avoidance in the physical system. A cosine-based oscillating bound B is defined (90° phase-shifted from *A*) as defined in Equation (24)

(24) $$B=\mathrm{UB}-\left|\frac{\mathrm{UB}\cdot t\cdot \cos\left(2\pi r\right)}{T}\right|$$

The phase shift ensures that rolling (using *A*) and ejection (using *B*) search different regions of the solution space. The ejection mechanism requires additional physics parameters as defined in Equation (25)

(25) $$\begin{array}{ll} k=0.5+0.5r & \;(\mathrm{elasticity}\;\mathrm{coefficient})\;\\ x=3r/D & \;(\mathrm{awn}\;\mathrm{compression}\;\mathrm{length})\;\\ \theta =\pi r & \;(\mathrm{ejection}\;\mathrm{angle})\;\\ \alpha =\frac{1}{\pi }\exp\left(\frac{r^{\prime }}{T}\right) & \;(\mathrm{air}\;\mathrm{resistance}\;\mathrm{coefficient})\; \end{array}$$

where $r\in \left[0,\;1\right]$ is a random number, and $r^{\prime }$ is a random integer in $\left[0,t\right]$. The elasticity coefficient *k* models the spring constant of the awn, *x* represents the compression distance (stored energy is proportional to *x*^2^), *θ* determines the projectile trajectory, and *α* captures air resistance that increases with iteration count. The ejection displacement *J* is derived from classical projectile motion, as given in Equation (26)

(26) $$J=\frac{2kx^{2}\sin\left(2\theta \right)}{mg}\cdot \frac{r_{\dim}\left(-B,B\right)}{D}\cdot \left(1-\alpha \right)$$

where g=9.8/D is a scaled gravitational constant. The term $\sin\left(2\theta \right)$ maximizes range at $\theta =45^{\circ }$, and the factor (1 − *α*) dampens displacement as air resistance accumulates. The final position update for ejection, parallel to the rolling mechanism, is defined in Equation (27)

(27) $$X_{t}\left(i\right)=X_{\mathrm{best}\;}+J+c\cdot L\;é\mathrm{vy}\;\left(D\right)⊗X_{\mathrm{best}\;}$$

### 3.3. Stagnation-Aware Aquila Optimizer (SAAO)

The SAAO integrates the Aquila Optimizer with Animated Oat Optimization, augmented by three novel control mechanisms. The integration addresses two primary failure modes of the AO: (i) premature convergence caused by inadequate exploration diversity and (ii) persistent stagnation of individual agents even when nominally assigned to exploration. THE SAAO is built on three principles:

(1) Operator Diversity: six update rules (three exploration and three exploitation) selected uniformly at random, ensuring no single operator dominates;
(2) Stagnation Awareness: real-time monitoring at both global and individual levels with adaptive response mechanisms;
(3) Physics-Grounded Escape: rolling and ejection mechanisms from AOO that provide physically interpretable escape pathways.

#### 3.3.1. Motivation and Design Rationale

The two baseline algorithms address search balance in fundamentally different ways: the AO uses a deterministic temporal threshold to switch phases globally, while AOO uses a probabilistic gate ($r_{1}>0.5$) to partition exploration and exploitation randomly each iteration. Both strategies apply uniformly to all agents, ignoring the heterogeneous improvement histories that individual agents accumulate. An agent that has improved its fitness in the last iteration requires a different behavioral policy than an agent that has not improved for 50 consecutive iterations. Applying the same global phase decision to both is a structural inefficiency that the SAAO is specifically designed to eliminate. The fusion also targets operator complementarity. The AO’s X1 rule (Equation (4)) mixes best-solution guidance with mean-position drift, a hybrid signal that lacks a clear behavioral identity. AOO’s centroid-guided and elite-guided dispersal operators (Equation (18)) provide the same two reference signals but cleanly separated, allowing the algorithm to sample either population-level or best-solution level guidance at each step. In exploitation, the AO’s X3 rule involves complex mixed terms; AOO’s rolling and ejection operators tie displacement magnitude to individual physical properties, creating natural heterogeneity. The six-operator library of the SAAO inherits the best behavioral characteristics of each parent.

#### 3.3.2. Initialization and Parameter Setup

The SAAO initializes population $X\in {\mathbb{R}}^{N\times D}$ using Equations (1) and (2) from Section 3.1. Each agent *i* is assigned AOO physics parameters $\left\{m_{i},L_{i},e_{i}\right\}$ per Equation (16). Two stagnation counters are initialized: per-agent counters noImprove $\left[i\right]=0$ for i=1,…,N, and a global counter *g* = 0. At each iteration *t*, the SAAO computes: the cubic decay factor *c* via Equation (16); AO parameters $G_{1},G_{2},QF$ via Equations (12) and (13); sinusoidal bounds *A*, *B* via Equations (19) and (24); spiral coordinates via Equations (6)–(8); and the population centroid as defined in Equation (3).

#### 3.3.3. Adaptive Exploration Probability (AEP)

The first novel mechanism adjusts exploration probability based on global stagnation *g*, as expressed in Equation (28)

(28) $$P_{\mathrm{explore}\;}=\left\{\begin{array}{ll} 0.7, & \;\mathrm{if}\;g>50\\ 0.6, & \;\mathrm{if}\;g>20\\ 0.5+0.3\left(1-\frac{t}{T}\right), & \;\mathrm{otherwise}\; \end{array}\right.$$

Under normal conditions $P_{\mathrm{explore}\;}$ decays from 0.8 to 0.5. Moderate stagnation (20<g≤50) elevates it to 0.6; severe stagnation (g>50) raises it to 0.7. As implemented and evaluated, *P_explore_* (Equation (28)) is a global-stagnation-responsive quantity that summarizes how long the run has failed to improve the global best and characterizes the current search regime. The per-agent decision to enter exploration or exploitation is taken by Equation (29), which is governed by the temporal threshold *t* ≤ 2*T*/3 and the individual stagnation counter *noImprove[i]*; the operative adaptive departure from the AO’s fixed schedule is therefore delivered by the individual-level stagnation control (ISC), which forces exploration re-entry for any stagnant agent regardless of the global clock. Regarding the step structure of Equation (28), the three-tier rule produces discontinuous jumps in *P_explore_* at g = 20 and g = 50. These jumps raise the exploration tendency only after sustained global stagnation and are applied to a population in which most agents are already governed by the temporal/ISC gate, so abrupt behavioral switching was not observed in practice; nevertheless, a smooth, monotone schedule would remove the boundary discontinuities.

#### 3.3.4. Individual-Level Stagnation Control (ISC)

The second mechanism monitors each agent independently, as expressed in Equation (29)

(29) $$\mathrm{Phase}\left(i\right)=\left\{\begin{array}{ll} \;\mathrm{Exploration},\; & \;\mathrm{if}\;\left.\left(t\leq \frac{2T}{3}\right)\;\mathrm{OR}\;(\mathrm{noImprove}\;\left[i\right]>\tau_{\mathrm{ISC}\;}\right)\\ \;\mathrm{Exploitation},\; & \;\mathrm{otherwise}\; \end{array}\right.$$

where $\tau_{\mathrm{ISC}\;}=30$. Trapped agents are redirected to exploration regardless of the global phase. The stagnation thresholds $\tau_{\mathrm{ISC}\;}$= 30 and $\tau_{\mathrm{OBR}}$ = 100 (Section 3.3.8) were selected empirically as the values at which an agent is, respectively, persistently and severely stagnant. The intent is a clear separation of timescales relative to the run length: $\tau_{\mathrm{ISC}\;}$ is short enough that an unproductive agent is redirected to exploration well before the run ends, while $\tau_{\mathrm{OBR}}$ is roughly three times larger so that the more disruptive full reinitialization is reserved for agents that remain stuck even after repeated exploration attempts. These constants are intentionally held fixed (and independent of dimension and population size) so that the SAAO introduces no problem-specific tuning, consistent with the No Free Lunch caution discussed in Section 1.

#### 3.3.5. Exploration Phase: Three Modes

When agent *i* enters exploration, one mode is selected uniformly (probability $\frac{1}{3}$ each). Mode E1 (Centroid-Guided): Using perturbation *W* from Equation (17), this is expressed in Equation (30)

(30) $$X_{\mathrm{new},i}=X_{\mathrm{centroid}\;}+W$$

Mode E2 (Elite-Guided) is defined in Equation (31)

(31) $$X_{\mathrm{new},i}=X_{\mathrm{best}}+W$$

Mode E3 (Lévy with Spiral) is achieved using spiral coordinates from Equations (6)–(8), expressed in Equation (32)

(32) $$X_{\mathrm{new},i}=X_{\mathrm{best}\;}\cdot \;Lé\mathrm{vy}\;\left(D\right)+X_{\mathrm{rand}\;}+\left(y^{\mathrm{sp}\;}-x^{\mathrm{sp}\;}\right)\cdot \mathrm{rand}()$$

#### 3.3.6. Exploitation Phase: Three Modes

When agent *i* enters exploitation, one mode is selected uniformly. Mode X1 (Rolling): Using displacement *R* from Equation (20), this is expressed in Equation (33)

(33) $$X_{\mathrm{new},i}=X_{\mathrm{best}\;}+R+c\cdot \;Lé\mathrm{vy}\;\left(D\right)⊗X_{\mathrm{best}\;}$$

Mode X2 (Ejection) is achieved using displacement *J* from Equation (26), as expressed in Equation (34)

(34) $$X_{\mathrm{new},i}=X_{\mathrm{best}\;}+J+c\cdot \;Lé\mathrm{vy}\;\left(D\right)⊗X_{\mathrm{best}\;}$$

Mode X3 (QF-based) is achieved using the quality function from Equations (11)–(13), as expressed in Equation (35)

(35) $$X_{\mathrm{new},i}=QF\cdot X_{\mathrm{best}\;}-G_{2}\cdot X_{i}\cdot \mathrm{rand}()-G_{1}\cdot \;Lé\mathrm{vy}\;\left(D\right)+\mathrm{rand}()\cdot G_{2}$$

#### 3.3.7. Selection and Stagnation Update

Greedy selection updates the population as defined in Equation (36)

(36) $$\left(X_{i},f_{i},\;\mathrm{noImprove}\;\left[i\right]\right)\leftarrow \left\{\begin{array}{ll} \left(X_{\mathrm{new},i},f\left(X_{\mathrm{new},i}\right),0\right), & \;\mathrm{if}\;f\left(X_{\mathrm{new},i}\right)<f_{i}\\ \left(X_{i},f_{i},\;\mathrm{noImprove}\;\left[i\right]+1\right), & \;\mathrm{otherwise}\; \end{array}\right.$$

#### 3.3.8. Opposition-Based Reinitialization (OBR)

The third mechanism addresses severely stagnant agents (noImprove $\left[i\right]>\tau_{\mathrm{OBR}}=100$). Random reinitialization is given in Equation (37)

(37) $$X_{i}=\mathrm{rand}\left(D\right)\cdot \left(\mathrm{UB}-\mathrm{LB}\right)+\mathrm{LB}$$

Opposition-based learning (OBL) is defined in Equation (38)

(38) $$X_{i}=\mathrm{LB}+\mathrm{UB}-X_{\mathrm{best}\;}+0.1\cdot \mathrm{randn}\left(D\right)\cdot \left(\mathrm{UB}-\mathrm{LB}\right)$$

Each strategy is selected with probability = 0.5. After reinitialization, fitness is evaluated, and noImprove [*i*] resets to zero. Two clarifications regarding Equation (38) are warranted. First, classical opposition-based learning reflects a solution about the center of the search range, which for a stagnant agent X_i_ would reproduce information already known to be unproductive. We therefore construct the opposite point about the current global best X_best_ rather than about X_i_: this relocates a severely stagnant agent into the region diametrically opposite the best-known attractor, which empirically provides a more useful escape direction than reflecting an already-trapped position, while the additive Gaussian term 0.1·*randn*(*D*)·(*UB* − *LB*) prevents all reinitialized agents from collapsing onto a single deterministic opposite point. Second, the Gaussian perturbation can, in principle, push a candidate outside [LB, UB]; in the implementation, the resulting vector is element-wise clipped back into the feasible box immediately after Equation (38) and before fitness evaluation, so no infeasible candidates are produced.

#### 3.3.9. Global Stagnation Tracking

**Figure 1:**
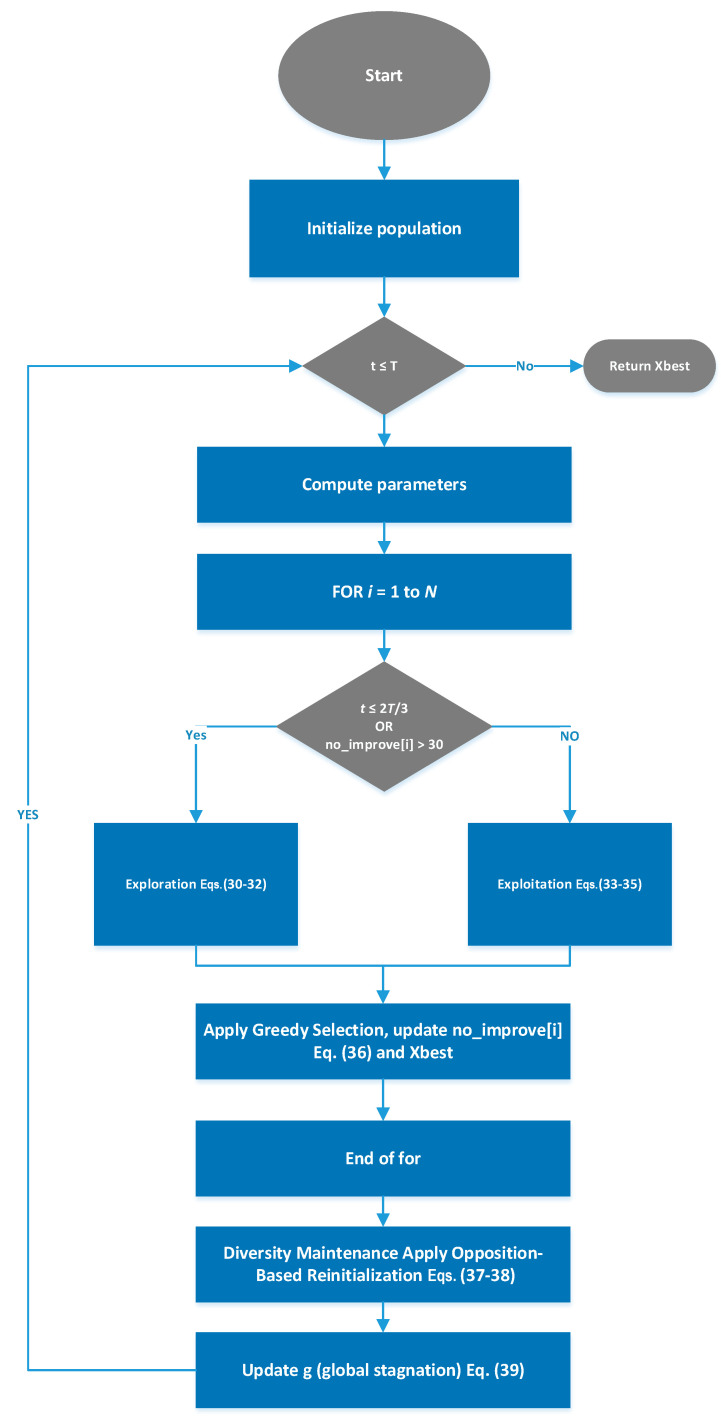
Flow chart of SAAO.

At each iteration’s end, the global stagnation tracking updated as follows in Equation (39)

(39) $$g\leftarrow \left\{\begin{array}{l} g+1\;\;\mathrm{if}\;\left|F_{\mathrm{best}\;}-F_{\mathrm{prev}\;}\right|<\varepsilon \\ 0\;\;\;\;\mathrm{otherwise}\; \end{array},F_{\mathrm{prev}\;}\leftarrow F_{\mathrm{best}\;}\right.$$

where $\varepsilon =10^{-10}$. Counter *g* feeds into AEP (Equation (28)). The pseudocode of the SAAO is presented in Algorithm 1, and the flow chart is presented in Figure 1. The source of the SAAO is available at https://github.com/MetaHeuLab/SAAO accessed on 3 July 2026.
**Algorithm**  **1.** Pseudocode of SAAO$\mathrm{Objective}\;F,\;\mathrm{bounds}\;{\left[\mathrm{LB},\mathrm{UB}\right]}^{D},\;\mathrm{population}\;\mathrm{size}\;N,\;\mathrm{iterations}\;T,\tau_{\mathrm{ISC}}=30,\tau_{\mathrm{OBR}}=100$ $\mathrm{Initialize}\;X\;\mathrm{via}\;\mathrm{Equations}\;(1)\;\mathrm{and}\;(2);\;\mathrm{assign}\;\mathrm{physics}\;\mathrm{params}\;\left\{m_{i},L_{i},e_{i}\right\}\;\mathrm{via}\;\mathrm{Equation}\;(16)$ $\mathrm{noImprove}\;\left[1:N\right]\leftarrow 0;g\leftarrow 0$ $\mathrm{Evaluate}\;f\left(X_{i}\right)\;\mathrm{for}\;\mathrm{all}\;i;\;\mathrm{set}\;X_{\mathrm{best}\;},F_{\mathrm{best}\;};F_{\mathrm{prev}\;}\leftarrow F_{\mathrm{best}\;}$ for t←1 to T do     $\mathrm{Compute}\;c,G_{1},G_{2},QF,A,B,\;\mathrm{spiral}\;\mathrm{coords},\;X_{\mathrm{centroid}\;}$    $P_{\mathrm{explore}\;}\leftarrow \mathrm{AEP}\left(g,t,T\right)$    for i←1 to N do      $\;\mathrm{if}\;\left(t\leq \frac{2T}{3}\right)\;\mathrm{or}\;\left(\;\mathrm{noImprove}\;\left[i\right]>\tau_{\mathrm{ISC}}\right)\;\mathrm{then}\;$           $X_{\mathrm{new}\;}\leftarrow \;\mathrm{SelectMode}(E1\;|\;E2\;|\;E3)\;▹\;\mathrm{Equations}\;(30)–(32)\;$     Else           $X_{\mathrm{new}\;}\leftarrow \;\mathrm{SelectMode}\;\left(X1\left|X2\right|X3\right)▹\;\mathrm{Equations}\;(33)–(35)\;$     end if       $\mathrm{Evaluate}\;f\left(X_{\mathrm{new}\;}\right);\;\mathrm{update}\;(X_{i},f_{i},\;\mathrm{noImprove}\;\left[i\right])\;\mathrm{via}\;\mathrm{Equation}\;(36)$     $\mathrm{if}\;f(X_{\mathrm{new}\;})<F_{\mathrm{best}\;}\;\mathrm{then}\;$        $F_{\mathrm{best}\;}\leftarrow f\left(X_{\mathrm{new}\;}\right);X_{\mathrm{best}\;}\leftarrow X_{\mathrm{new}\;}$     **end if**    **end for**    for i←1 to N do      $\mathrm{if}\;\mathrm{noImprove}\;\left[i\right]>\tau_{\mathrm{OBR}}\;\mathrm{then}\;$            With prob. 0.5: random reinit (Equation (37)); else OBL reinit (Equation (38))            $\mathrm{Evaluate}\;f\left(X_{i}\right);\;\mathrm{noImprove}\;\left[i\right]\leftarrow 0$
           $\mathrm{if}\;f(X_{i})<F_{\mathrm{best}\;}\;\mathrm{then}\;F_{\mathrm{best}\;}\leftarrow f(X_{i});X_{\mathrm{best}\;}\leftarrow X_{i}$
           **end if**
      **end if**
    **end for**
    $\mathrm{Update}\;g\;\mathrm{via}\;\mathrm{Equation}\;(39);\;F_{\mathrm{prev}\;}\leftarrow F_{\mathrm{best}\;}$
 **end for**
 $\mathrm{return}\;X_{\mathrm{best}\;},F_{\mathrm{best}\;}$

### 3.4. Computational Complexity

The SAAO’s inner loop evaluates one candidate per agent per iteration: $O\left(N\right)$ evaluations, each requiring $O\left(D\right)$ operator computation plus *O*(COF) objective function cost. The diversity maintenance step adds $O\left(N\right)$ counter comparisons and at most $O\left(N\right)$ reinitializations, each costing *O*(*D* + COF). No population-wide pairwise computations are performed. The global stagnation counter and individual counters add $O\left(N\right)$ bookkeeping. Total time complexity is therefore as defined in Equation (40)

(40) $$T_{\mathrm{SAAO}}=O\left(T\cdot N\cdot \left(D+\mathrm{COF}\right)\right)$$

identical in asymptotic order to the original AO and AOO. Space complexity is $O\left(N\cdot D+T\right)$ for storing population positions and the convergence curve. The SAAO adds $O\left(N\right)$ space for the per-agent counter vector *η*, which is negligible.

## 4. Experiment and Discussion

### 4.1. Experimental Setup

Two standard benchmark suites are used: CEC2015 [36], comprising F1–F15 in 30 dimensions (F1–F2 unimodal, F3–F5 simple multimodal, F6–F8 hybrid, an dF9–F15 composition), and CEC2022, comprising F16–F27 in 20 dimensions (F16 unimodal, F17–F20 basic multimodal, F21–F23 hybrid, and F24–F27 composition) [37,38]. All functions are evaluated over the search space ${\left[-100,100\right]}^{D}$. All algorithms used a population size of 30, maximum iterations of 6000, and the same search bounds. Comparison algorithms: Nine state-of-the-art algorithms serve as baselines: Aquila Optimizer (AO) [32], Animated Oat Optimization Algorithm (AOO) [21], Black Kite Algorithm (BKA) [39], Crayfish Optimization Algorithm (COA) [40], Dung Beetle Optimizer (DBO) [41], Parrot Optimizer (PO) [42], Pelican Optimization Algorithm (POA) [43], Tasmanian Devil Optimization (TDO) [44], and Transient search algorithm (TSO) [45]; the parameters of these algorithms are presented in Table 1. Each algorithm is run 30 times per function. Results are reported as the mean (AVG) and standard deviation (STD). Statistical significance is assessed via the Wilcoxon rank-sum test (applied across functions) at α=0.05 and the Friedman mean-rank test.

### 4.2. Comparison on CEC2015 and CEC2022 Benchmark Functions

Table 2 reports the mean and standard deviation for 30 independent runs on CEC2015. The SAAO achieves the best mean on 11 of 15 functions (F1, F3, F4, F5, F6, F7, F8, F9, F10, F12, and F15) and the best standard deviation on 8 functions, indicating both accuracy and consistency. The empirical evaluation demonstrates that the proposed SAAO exhibits superior convergence characteristics across multiple benchmark categories. On the unimodal function F1, the SAAO attains a mean objective value of 2.093×10^3^, representing a significant improvement to the standard AO (1.661 × 10^7^) and AOO (4.136 × 10^3^). This substantial performance differential underscores the efficacy of the integrated stagnation control mechanism in mitigating premature convergence, a prevalent failure mode when navigating narrow, ill-conditioned search valleys.

For the hybrid composition function F8, the SAAO yields a mean result of 8.035 × 10^2^, surpassing the BKA (3.785 × 10^7^). Notably, competing algorithms, including TSO, the COA, DBO, PO, and the POA, exhibit high standard deviations across independent runs, suggesting a pronounced susceptibility to entrapment in disparate local optima. In contrast, the SAAO maintains both solution quality and stability. On the more complex composition functions F12 and F15, the SAAO consistently achieves the lowest mean values. Moreover, on F12, it concurrently records the minimal standard deviation, thereby affirming that the incorporation of OBL for diversity preservation effectively curbs the extreme run-to-run variability observed in alternative approaches. For instance, the BKA and TSO report higher standard deviations, respectively, on F12 values that reflect an inconsistent exploration–exploitation balance. It should be acknowledged that the SAAO does not uniformly dominate across all test instances. On functions F11 and F14, TDO attains marginally superior mean values. On F11, TDO’s operator appears advantageous for navigating composition landscapes with widely dispersed optima, albeit at the cost of elevated variance. Conversely, while the SAAO’s mean on F11 is slightly higher, it demonstrates enhanced robustness on F14, with a standard deviation of 6.381 × 10^1^ compared to TDO’s 7.397 × 10^1^. This pattern suggests that the SAAO prioritizes convergence consistency over aggressive, high-variance search behavior, a trade-off that may prove beneficial in applications demanding reliable, repeatable performance.

Performance evaluation across the CEC2022 benchmark suite indicates that the SAAO maintains a competitive optimization profile, securing the lowest or jointly lowest mean objective value across eight of the twelve test functions (F16, F18, F20, F22, F23, F25, F26, and F27), as seen in Table 3. Concurrently, the algorithm demonstrates superior convergence stability, recording the minimal standard deviation. This dual emphasis on solution precision and run-to-run consistency suggests that the integrated stagnation-alleviation framework effectively modulates the exploration–exploitation trade-off, particularly within high-dimensional, multimodal search environments. An isolated deviation occurs on the basic multimodal function F17, where AOO yields a marginally lower mean value (4.449 × 10^2^ versus 4.480 × 10^2^ for the SAAO). Despite this slight reduction in average solution quality, the SAAO exhibits markedly enhanced robustness, achieving the lowest standard deviation (1.885 × 10^0^). This outcome implies that while opposition-based initialization may occasionally facilitate the discovery of marginally deeper optima, the SAAO’s adaptive control mechanism prioritizes convergence reliability.

Comparative analysis reveals that TDO outperforms the SAAO on functions F19, F21, F24, and F26. This performance distribution aligns with TDO’s scavenging-inspired search dynamics, which prove particularly advantageous when the global optimum resides within a geometrically isolated attraction basin. In such topological configurations, localized exploitation strategies can rapidly isolate promising regions without being prematurely diverted by adjacent suboptimal structures. However, this specialized strength does not generalize uniformly across more complex fitness landscapes, as evidenced by TDO’s comparative decline on interleaved composition functions where basin boundaries are less distinct. A particularly instructive contrast emerges on the composition function F25, where the SAAO attains a mean objective value of 2.786 × 10^3^, substantially outperforming AOO’s 3.471 × 10^3^. This represents a clear inversion of their relative rankings on the CEC2015 benchmark set and highlights a fundamental shift in search dynamics attributable to the SAAO’s stagnation control architecture. Composition landscapes characterized by interleaved suboptimal basins of heterogeneous depth routinely mislead purely physics-mimicking operators, which typically lack the adaptive feedback mechanisms required to recognize and escape deceptive gradient-like cues. By continuously modulating exploration intensity in response to population stagnation metrics, the SAAO circumvents these entrapment pathways, enabling sustained progression through highly deceptive topographies. This behavioral adaptation underscores the algorithm’s capacity to maintain directional momentum in environments where static or purely stochastic search strategies routinely degrade, offering a more resilient framework for complex, real-world optimization tasks.

### 4.3. Convergence and Behavioral Analysis

**Figure 2:**
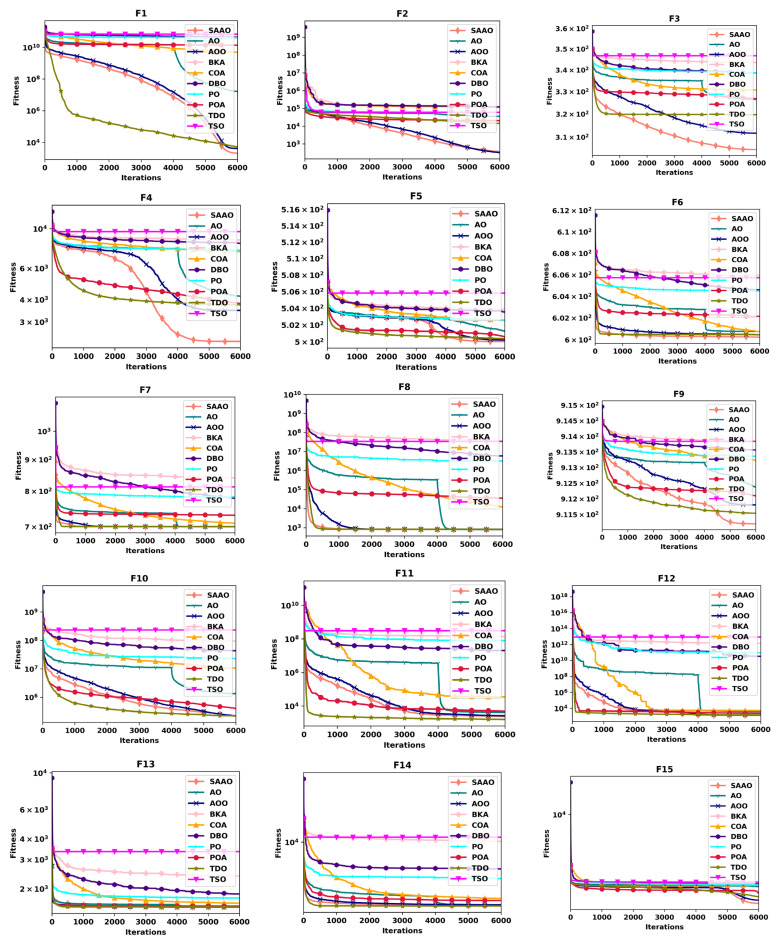
Convergence curves of SAAO and compared optimizers on CEC2015 (F1-F15).

**Figure 3:**
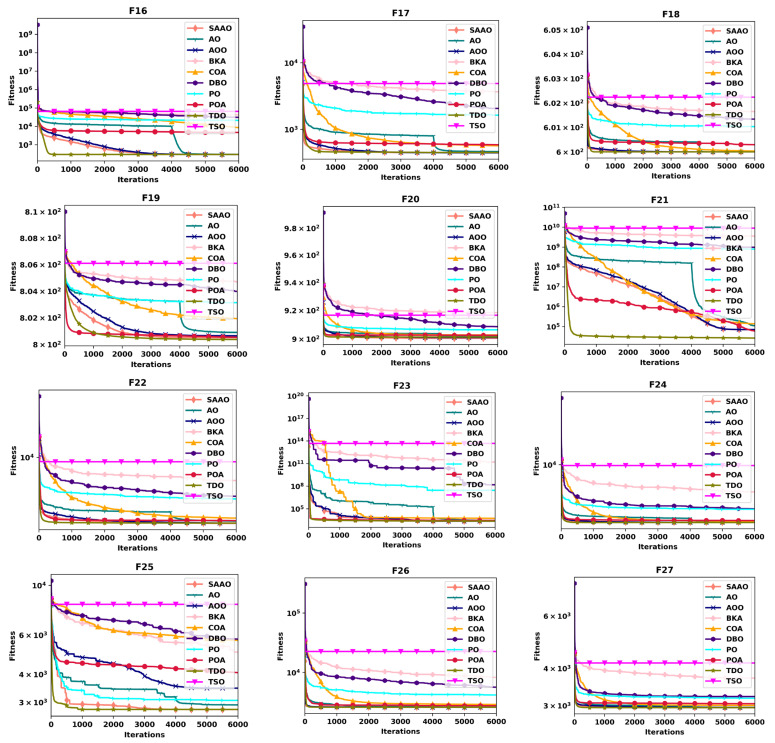
Convergence curves of SAAO and compared optimizers on CEC2022.

**Figure 4:**
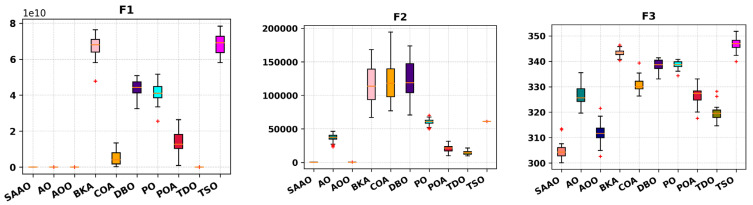

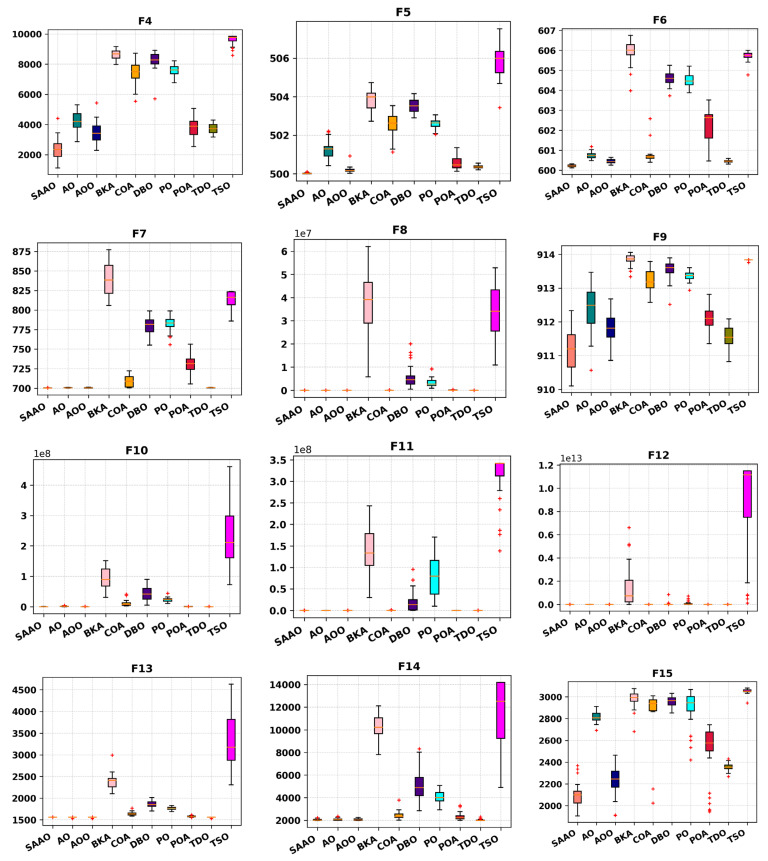
Box plots of SAAO and compared optimizers on CEC2015.

**Figure 5:**
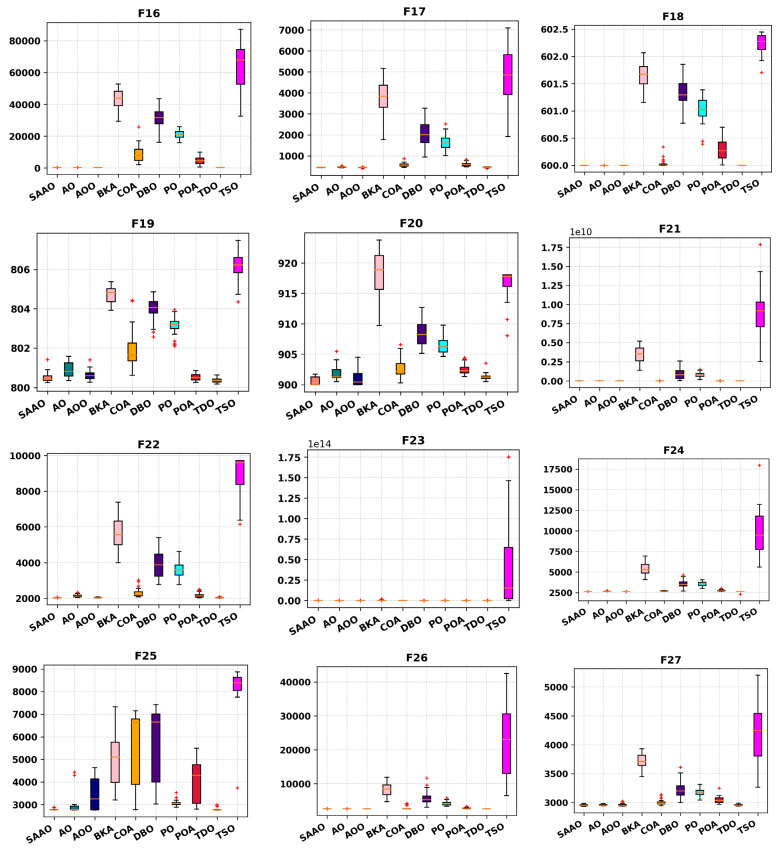
Box plots of SAAO and compared optimizers on CEC2022.

Convergence curves (Figure 2 and Figure 3) reveal three characteristic convergence signatures associated with the SAAO’s control mechanisms. On functions where exploration is critical, the SAAO’s convergence curve drops sharply in the first 1000 iterations before settling into a steady refinement trajectory. This early drop is attributable to the high initial $P_{\mathrm{explore}\;}$ value (0.80 at *t* = 0), which sustains broad sampling while other algorithms begin premature exploitation. The AO, which uses a fixed 2/3 threshold, begins exploitation at iteration 4000 (of 6000) regardless of population diversity; the SAAO’s adaptive mechanism allows continued exploration past this point when stagnation is detected. On composition functions, the SAAO achieves lower asymptotic fitness than competitors with smaller final variance. The diversity maintenance module prevents population collapse in late iterations when multiple agents would otherwise concentrate at the same local optimum, maintaining the effective search radius needed to escape shallow traps. The box plot distribution of the CEC2015 and CEC2022 functions is presented in Figure 4 and Figure 5.

**Figure 6:**
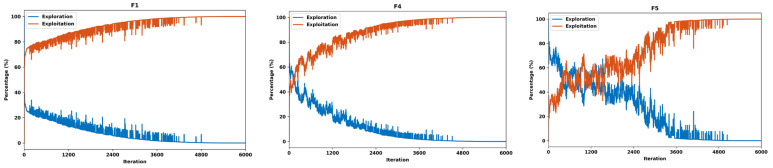

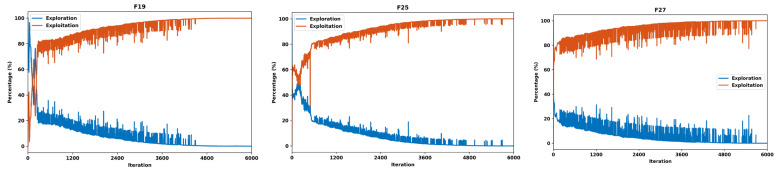
Exploration vs. exploitation plots of SAAO.

**Figure 7:**
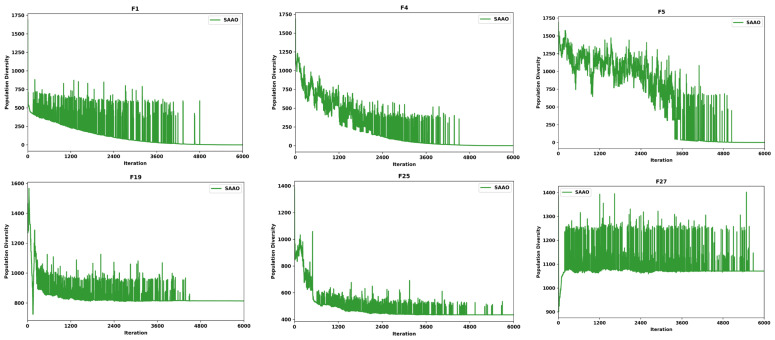
Diversity plots of SAAO.

**Figure 8:**
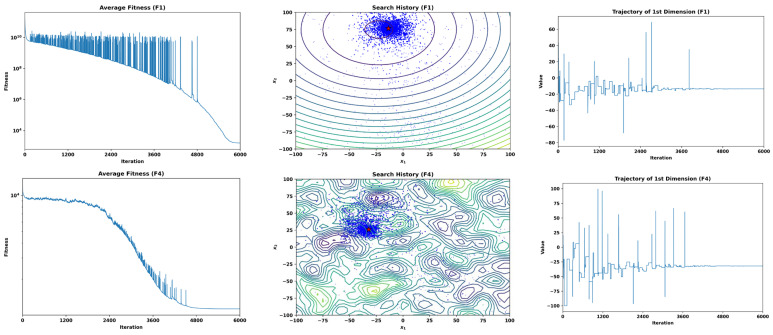

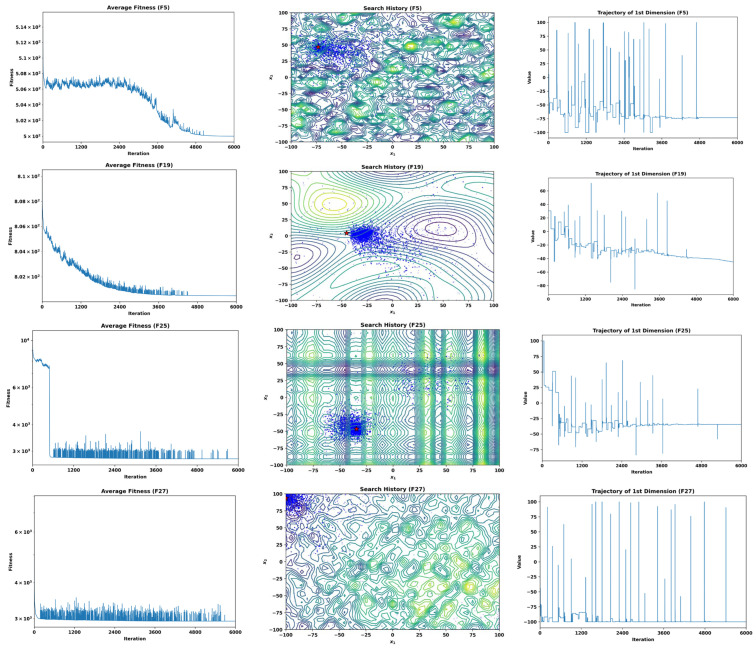
Qualitative performance of SAAO on CEC2015 and CEC2022.

Exploration–exploitation balance plots (Figure 6) confirm that the SAAO maintains higher exploration ratios beyond iteration 4000 on stagnation-prone functions, while maintaining exploitation intensity on functions where the landscape is regular. Population diversity plots (Figure 7) show that the SAAO’s diversity degrades more slowly on composition functions, with diversity level maintained by the reinitialization module. Qualitative performance plots (Figure 8) display the average fitness, search history, and first-dimension trajectory for selected functions. The search history confirms the two-phase character: a broad scattered pattern in early iterations transitions to a concentrated cluster with occasional distant excursions, corresponding to the ejection mechanism’s periodic far-field jumps.

### 4.4. Statistical Analysis

All *p*-values are in Table 4, with the *α* = 0.05 significance threshold except SAAO vs. TDO on CEC2022 (*p* = 0.477), indicating that the SAAO’s advantage over TDO on that suite is not statistically significant at the tested level, a finding consistent with TDO’s strong per-function performance noted in Section 4.1. All other pairwise comparisons confirm the SAAO’s superiority at *p* < 0.05, with the majority at *p* < 0.01. The Friedman mean-rank test assigns the SAAO rank 1 on both benchmark suites (1.367 on CEC2015 and 1.792 on CEC2022), making it the only algorithm that holds the top rank across both test environments. On CEC2015, AOO earns Friedman rank 2 (2.167), confirming that the AOO components contribute meaningfully to the SAAO’s performance. On CEC2022, TDO earns Friedman rank 2 (4.958), ahead of the SAAO’s parent algorithms (the AO and AOO). This cross-suite ranking stability is a critical indicator of generalization. The SAAO’s overall superiority does not depend on benchmark-specific characteristics. In Table 4, the test used throughout is the Wilcoxon rank-sum test; it was applied across functions, comparing, for each competitor, the per-function results of the SAAO against that competitor over all functions of a suite.

### 4.5. Engineering Problem

#### 4.5.1. I-Shaped Beam

The structural configuration of an I-shaped beam is formulated as a continuous optimization problem targeting the minimization of vertical deflection under static loading. The mathematical model is defined in Equation (41) [46]. The design vector comprises four continuous variables representing cross-sectional dimensions: flange width $\left(x_{1}\right)$, overall section height $\left(x_{2}\right)$, web thickness $\left(x_{3}\right)$, and flange thickness $\left(x_{4}\right)$. The objective function is inversely proportional to the second moment of area, thereby establishing a direct relationship between the minimization of $f\left(X\right)$ and enhancement of bending stiffness. Two nonlinear inequality constraints govern structural feasibility: $g_{1}\left(X\right)$ imposes an upper bound on the material cross-sectional area, while $g_{2}\left(X\right)$ restricts the maximum bending stress to a permissible engineering threshold. Variable bounds reflect standard manufacturing tolerances and buckling stability requirements.

(41) $$\min f\left(X\right)=\frac{500}{\frac{x_{3}{\left(x_{2}-2x_{4}\right)}^{3}}{12}+\frac{x_{1}x_{4}^{3}}{6}+2bx_{4}{\left(x_{2}-x_{4}\right)}^{2}}$$

Subject to:

(42) $$\begin{array}{l} g_{1}\left(X\right)\;=2x_{1}x_{3}+x_{3}\left(x_{2}-2x_{4}\right)\leq 300\\ g_{2}\left(X\right)\;=\frac{18x_{2}\times 10^{4}}{x_{3}{\left(x_{2}-2x_{4}\right)}^{3}+2x_{1}x_{3}\left[4x_{4}^{2}+3x_{2}\left(x_{2}-2x_{4}\right)\right]}+\frac{15x_{1}\times 10^{3}}{\left(x_{2}-2x_{4}\right)x_{3}^{2}+2x_{3}x_{1}^{3}}\leq 56 \end{array}$$

Variable bounds:

(43) $$10\leq x_{1}\leq 50,\;10\leq x_{2}\leq 80,\;0.9\leq x_{3}\leq 5,\;0.9\leq x_{4}\leq 5$$

#### 4.5.2. Tubular Column Design

This benchmark addresses the cost-optimal sizing of a cylindrical column subjected to axial compressive loading. The design space is parameterized by two continuous variables: the mean outer diameter ($k_{1}$) and the wall thickness ($k_{2}$). The objective function, specified in Equation (45), models a linear approximation of material procurement and fabrication expenses. Structural integrity is maintained through six inequality constraints $\left(g_{1}-g_{6}\right)$ that collectively enforce buckling resistance, yield strength limits, stress thresholds, and geometric feasibility. The admissible search domain is restricted to standard industrial specifications, ensuring that all candidate solutions remain within practical manufacturing limits.

Design vector:

(44) $$\vec{k}=\left[k_{1},k_{2}\right]=\left[d,t\right]$$

Minimize:

(45) $$f\left(\vec{k}\right)=9.8k_{1}k_{2}+2k_{2}$$

Variable bounds:

(46) $$2\leq k_{1}\leq 14,\;0.2\leq k_{2}\leq 0.8$$

Subject to:

(47) $$\begin{array}{l} g_{1}\left(\vec{k}\right)=1.59-k_{1}k_{2}\leq 0\\ g_{2}\left(\vec{k}\right)=47.4-k_{1}k_{2}\left(k_{1}^{2}+k_{2}^{2}\right)\leq 0\\ g_{3}\left(\vec{k}\right)=\frac{2.0}{k_{1}}-1\leq 0\\ g_{4}\left(\vec{k}\right)=\frac{k_{1}}{14}-1\leq 0\\ g_{5}\left(\vec{k}\right)=\frac{0.2}{k_{2}}-1\leq 0\\ g_{6}\left(\vec{k}\right)=\frac{k_{2}}{8}-1\leq 0 \end{array}$$

#### 4.5.3. Gear Train Design Problem

The design of a multi-stage gear train constitutes a discrete combinatorial optimization problem focused on achieving a target velocity ratio while minimizing component volume and associated manufacturing costs. The transmission system comprises four meshing gears (A, B, D, and F). The decision vector consists of the integer tooth counts for each gear: $T_{a}\left(k_{1}\right),T_{b}\left(k_{2}\right),T_{d}\left(k_{3}\right)$, and $T_{f}\left(k_{4}\right)$. The objective function quantifies the squared deviation between the realized transmission ratio and a prescribed target value. The non-convex, integer-constrained search space introduces significant algorithmic complexity, as optimal configurations must align with isolated feasible coordinates rather than continuous gradients. The mathematical formulation is defined in Equation (50)

Design vector:

(48) $$\vec{k}=\left[k_{1},k_{2},k_{3},k_{4}\right]=\left[T_{a},T_{b},T_{d},T_{f}\right]$$

Minimize:

(49) $$f\left(\vec{k}\right)={\left(\frac{1}{6.931}-\frac{T_{b}T_{d}}{T_{a}T_{f}}\right)}^{2}$$

Variable domain:

(50) $$k_{i}\in \left\{12,13,14,\ldots ,60\right\},\;i=1,2,3,4$$

In the IBD problem, the objective is to minimize the deflection of an I-shaped cross-section under a point load, subject to bending stress, shear stress, and geometry constraints. The SAAO achieves the best-known mean of 1.745821E-04, as seen in Table 5, tied with AOO, PO, the POA, and TSO. The extremely low variance confirms deterministic convergence to the constrained optimum across all 30 runs; all parameters are kept the same in the previous experiment. TDO exhibits the largest deviation, suggesting that its scavenging mechanism struggles when the feasible region is geometrically thin.

For the TCD problem, the objective minimizes the weight of a thin-walled hollow column subject to buckling and yielding constraints. The SAAO achieves 30.149740, matching the best-known value, with near-zero variance. AO and DBO exhibit means of 30.217960 and 30.21757, respectively, indicating convergence to a nearby local optimum. For the GTD problem, the objective minimizes the error between the achieved and required gear ratio, a discrete combinatorial problem embedded in a continuous search framework. The SAAO achieves a mean of 1.679E-20 versus AO’s, an improvement attributable to the ISC mechanism forcing exploration when the discrete gear ratio landscape traps agents at incorrect gear combinations. AOO achieves the best result (1.312E-24), confirming that the rolling and ejection operators from the physics framework are particularly effective on discrete-like landscapes with narrow precision requirements. All three engineering problems were solved using the open-source ENOPPY library [47], which supplies the objective function, the inequality and box constraints, and a penalty-based constraint-handling scheme; the optimizer only receives a scalar penalized objective and the variable bounds, so no problem-specific solver tuning is performed, and the variable bounds are taken directly from the library because feasibility is as important as the objective value. Finally, for the IBD and TCD problems, the SAAO ties with several modern optimizers (AOO, PO, POA, TSO for IBD; AOO, POA, TSO for TCD) at essentially the near best-known value with near-zero variance.

### 4.6. Real-World Case Study: Equipment Anomaly Prediction

**Figure 9:**
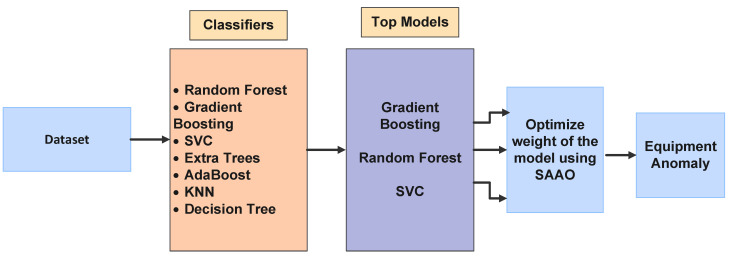
Ensemble-SAAO model.

The real-world validation uses an industrial equipment anomaly detection dataset comprising readings from machinery, with a binary label indicating normal versus anomalous operation [48]. Features include vibration amplitude, temperature, pressure, humidity, equipment type, and location. Preprocessing involved min–max normalization of the four numeric features (temperature, pressure, vibration, and humidity), with the scaler fitted on the training partition only; one-hot encoding of the two categorical features (equipment type and location); and removal of duplicate and missing-value rows. After cleaning, the dataset contains 7672 samples and six raw features that expand to eleven model inputs after one-hot encoding. Because anomaly detection data are imbalanced, accuracy alone can be misleading, so precision, recall, F1, AUC, and log-loss are also reported in Table 6. To avoid optimistic bias, the data are divided by stratified sampling into three disjoint partitions training, validation, and test; the SAAO ensemble weights are optimized exclusively on the validation partition, while the test partition is held out and used only for the final results in Table 6. Several classifiers serve as comparison baselines: Random Forest (RF), Gradient Boosting (GB), Support Vector Classifier (SVC), Extra Trees (ET), AdaBoost, K-Nearest Neighbors (KNN), and Decision Tree (DT). All baselines use default hyperparameters. The seven baselines were selected to span the principal families of supervised classifiers commonly used in industrial fault detection, so that the ensemble is assembled from diverse and complementary error profiles: bagging-based tree ensembles (Random Forest and Extra Trees), boosting-based tree ensembles (Gradient Boosting and AdaBoost), a margin-based kernel method (Support Vector Classifier), an instance-based method (K-Nearest Neighbors), and a tree-based model (Decision Tree). This spread of inductive biases is intentional because a weighted voting ensemble benefits most when its members make uncorrelated errors. The SAAO optimizes a voting ensemble of the three best models from the base models, where the optimization targets are the weight coefficients $w=\left(w_{1},w_{2},w_{3}\right)$ with $\sum w_{i}=1,w_{i}\in \left[0,1\right]$ [49]. The SAAO maximizes the $F_{1}^{\mathrm{weighted}\;}$, with 30 agents and 500 iterations; the model is presented in Figure 9. The SAAO-optimized ensemble achieves the highest accuracy (98.23%), the highest F1-score (0.9119), and the lowest log-loss (0.064) among the eight models in Table 6. It does not, however, dominate on every metric: its AUC (0.9805) is marginally below that of Gradient Boosting (0.9824), F1-score, and log-loss while remaining highly competitive on AUC. The low log-loss is consistent with well-calibrated probability estimates. The performances reported confirm that Ensemble-SAAO produces a well-calibrated model, a critical property in industrial anomaly detection.

## 5. Conclusions

This paper addressed two persistent failure modes in population-based metaheuristic optimization premature convergence driven by inadequate late-phase exploration diversity and agent stagnation that persists silently when phase-assignment decisions ignore individual improvement history. The Stagnation-Aware Aquila Optimizer (SAAO) was developed to address both gaps through three novel control mechanisms layered onto a hybrid operator architecture. The Adaptive Exploration Probability mechanism responds to real-time global stagnation signals, boosting exploration probability as stagnation deepens. The Individual-Level Stagnation Control mechanism maintains a per-agent non-improvement counter and redirects stagnant agents to exploration regardless of the global optimization phase, targeting corrective action precisely where needed. The Diversity Maintenance module reinitializes completely stagnant agents using opposition-based learning or uniform sampling, preventing population collapse in late-run iterations on composition and hybrid landscapes.

Across 27 benchmark functions spanning two independent CEC test suites—CEC2015 in 30 dimensions and CEC2022 in 20 dimensions— the SAAO achieved Friedman rank 1 on both suites, with Wilcoxon rank-sum tests applied across functions confirming statistically significant advantages over most of the compared algorithms; the advantage over TDO on CEC2022 was not significant. On the three engineering design problems, the SAAO matched or achieved the best-known value among the compared models. In the equipment anomaly detection application, the SAAO-optimized ensemble weighting produced the highest accuracy across seven baseline classifiers, demonstrating direct practical utility in an industrial monitoring context. Three directions are identified for future research. First, the individual stagnation threshold of 30 iterations and the reinitialization threshold of 100 iterations were set empirically; an automatic threshold adaptation rule—for example, one that rescales thresholds proportionally to problem dimensionality or to the observed improvement frequency in the current run—could further generalize the SAAO’s performance without introducing additional hyperparameters. Second, the SAAO’s operator library currently draws from two source algorithms; extending the library with operators from differential-mutation frameworks to adaptively allocate probability mass based on historical improvement rates could improve performance on problem classes not well-represented in CEC benchmarks. Beyond these two directions, this revision records several further limitations and planned extensions. (i) Ablation study: The three mechanisms (adaptive exploration probability, individual-level stagnation control, and opposition-based reinitialization) and the AOO-derived operators are tightly coupled; the stagnation counters feed both the phase gate and the reinitialization step, so disabling one mechanism alters the operating regime of the others, which makes a clean single-factor ablation non-trivial; a controlled component-wise ablation (the full SAAO versus the SAAO without AEP, without ISC, without OBR, and without the AOO operators) is planned as a dedicated study. (ii) Parameter sensitivity: the thresholds τ_ISC = 30 and τ_OBR = 100 were chosen empirically as an extreme case of stagnation; the sensitivity analysis, with a dimension and population-aware rescaling rule, will be investigated in future work. (iii) The global-stagnation criterion in Equation (39) may not trigger on functions whose optima have large magnitude, limiting the usefulness of the global counter there; a relative-tolerance criterion will be further investigated in the future. (iv) Statistical analysis: a multiple-comparison correction (Holm or Bonferroni adjustment, or a Nemenyi post hoc test after the Friedman ranking) will further be investigated. (v) Engineering and benchmarking scope: Only three low-dimensional engineering problems were used, on which most modern optimizers already perform near-optimally, and future work will add more and higher-dimensional engineering problems including biomimetic, and fluid/heat-transfer.

## Figures and Tables

**Table 1:** Parameter settings of compared optimizers.

Algorithm	Parameters
AO	µ = 0.00565, ω=0.005, α = δ = 0.1
AOO	β = 1.5
BKA	*P* = 0.9
COA	C_3_ = 3, μ = 25, σ = 3
DBO	P_percent = 0.2
PO	$\alpha =\mathrm{rand}\frac{\left[0,1\right]}{5}$, $\theta =\mathrm{rand}\left[0,1\right]*\pi$
POA	R = 0.2
TSO	$k=2,\;z\in \left[0,\;2\right]$
TDO	R = [0.01, 0]
SAAO	α = δ = 0.1, g = 9.8/Dim

**Table 2:** Result of SAAO and compared algorithms on CEC2015.

		SAAO	AO	AOO	BKA	COA	DBO	PO	POA	TDO	TSO
F1	AVG	**2.093E+3**	1.661E+7	4.136E+3	6.701E+10	4.877E+9	4.398E+10	4.125E+10	1.371E+10	5.331E+3	6.826E+10
	STD	**1.415E+3**	8.310E+6	7.661E+3	5.991E+9	3.773E+9	4.393E+9	5.358E+9	6.348E+9	1.363E+4	5.704E+9
F2	AVG	3.705E+2	3.611E+4	**3.279E+2**	1.150E+5	1.243E+5	1.218E+5	6.052E+4	2.050E+4	1.435E+4	6.126E+4
	STD	1.057E+2	6.240E+3	7.003E+1	2.760E+4	3.500E+4	2.859E+4	4.909E+3	5.630E+3	2.669E+3	**1.931E+1**
F3	AVG	**3.047E+2**	3.265E+2	3.116E+2	3.434E+2	3.307E+2	3.385E+2	3.386E+2	3.267E+2	3.197E+2	3.467E+2
	STD	3.054	3.508	3.935	1.448	2.845	2.252	**1.417**	3.409	2.731	2.513
F4	AVG	**2.334E+3**	4.185E+3	3.482E+3	8.644E+3	7.438E+3	8.269E+3	7.586E+3	3.808E+3	3.738E+3	9.623E+3
	STD	6.934E+2	7.232E+2	7.276E+2	**3.075E+2**	7.488E+2	5.944E+2	3.269E+2	6.062E+2	3.167E+2	3.344E+2
F5	AVG	**5.000E+2**	5.013E+2	5.002E+2	5.039E+2	5.025E+2	5.035E+2	5.026E+2	5.006E+2	5.004E+2	5.058E+2
	STD	**2.536E- 2**	4.372E-1	1.548E-1	5.285E-1	5.768E-1	3.364E-1	2.630E-1	3.495E-1	7.632E-2	8.975E-1
F6	AVG	**6.002E+2**	6.008E+2	6.004E+2	6.059E+2	6.008E+2	6.046E+2	6.045E+2	6.021E+2	6.005E+2	6.057E+2
	STD	**5.347E-2**	1.634E-1	1.031E-1	5.597E-1	4.124E-1	3.247E-1	3.191E-1	9.390E-1	8.028E-2	2.426E-1
F7	AVG	**7.003E+2**	7.005E+2	7.005E+2	8.402E+2	7.092E+2	7.787E+2	7.822E+2	7.309E+2	7.006E+2	8.124E+2
	STD	**1.224E-1**	2.120E-1	3.420E-1	2.123E+1	6.973	1.202E+1	9.100	1.113E+1	2.283E-1	1.168E+1
F8	AVG	**8.035E+2**	8.338E+2	8.062E+2	3.785E+7	1.232E+4	5.938E+6	3.252E+6	3.589E+4	8.230E+2	3.458E+7
	STD	**1.043**	9.047	1.637	1.448E+7	1.185E+4	4.922E+6	2.089E+6	4.892E+4	1.012E+1	1.126E+7
F9	AVG	**9.112E+2**	9.124E+2	9.118E+2	9.139E+2	9.132E+2	9.136E+2	9.134E+2	9.121E+2	9.115E+2	9.138E+2
	STD	6.453E-1	6.579E-1	4.941E-1	1.618E-1	3.204E-1	2.747E-1	1.368E-1	3.320E-1	3.428E-1	**1.469E-2**
F10	AVG	**2.170E+5**	1.346E+6	2.254E+5	9.490E+7	1.062E+7	4.353E+7	2.298E+7	3.968E+5	2.249E+5	2.345E+8
	STD	1.213E+5	8.034E+5	1.407E+5	3.286E+7	9.202E+6	2.185E+7	7.170E+6	2.442E+5	**8.574E+4**	1.100E+8
F11	AVG	2.441E+3	4.232E+3	2.712E+3	1.384E+8	3.391E+4	2.057E+7	7.997E+7	4.905E+3	**1.635E+3**	3.120E+8
	STD	3.093E+3	4.045E+3	3.375E+3	5.431E+7	1.522E+5	2.471E+7	4.710E+7	3.908E+3	**1.349E+3**	5.626E+7
F12	AVG	**1.370E+3**	3.967E+3	1.520E+3	1.449E+12	5.433E+3	3.311E+10	9.377E+10	2.515E+3	1.610E+3	8.754E+12
	STD	**1.498E+2**	9.800E+2	3.732E+2	1.740E+12	1.442E+3	1.550E+11	1.654E+11	8.425E+2	4.924E+2	4.163E+12
F13	AVG	1.559E+3	1.559E+3	**1.553E+3**	2.392E+3	1.638E+3	1.861E+3	1.763E+3	1.576E+3	1.557E+3	3.352E+3
	STD	**2.257E-3**	6.253	1.291E+1	1.909E+2	4.093E+1	7.985E+1	3.539E+1	1.288E+1	8.639	6.143E+2
F14	AVG	2.040E+3	2.081E+3	2.069E+3	1.020E+4	2.433E+3	5.112E+3	4.009E+3	2.309E+3	**2.014E+3**	1.134E+4
	STD	**6.381E+1**	1.026E+2	9.006E+1	1.099E+3	3.459E+2	1.388E+3	5.253E+2	3.098E+2	7.397E+1	3.237E+3
F15	AVG	**2.099E+3**	2.811E+3	2.223E+3	2.980E+3	2.878E+3	2.958E+3	2.894E+3	2.497E+3	2.355E+3	3.052E+3
	STD	1.087E+2	4.849E+1	1.493E+2	7.507E+1	2.203E+2	4.552E+1	1.648E+2	2.587E+2	3.452E+1	**2.340E+1**

**Table 3:** Results of SAAO and compared algorithms on CEC2022.

		SAAO	AO	AOO	BKA	COA	DBO	PO	POA	TDO	TSO
F16	AVG	**3.000E+2**	3.012E+2	**3.000E+2**	4.323E+4	8.740E+3	3.117E+4	2.108E+4	4.706E+3	**3.000E+2**	6.546E+4
	STD	5.285E-6	6.258E-1	2.434E-9	5.720E+3	5.257E+3	6.504E+3	2.562E+3	2.580E+3	**0**	1.546E+4
F17	AVG	4.480E+2	4.650E+2	**4.449E+2**	3.677E+3	5.678E+2	2.065E+3	1.636E+3	5.881E+2	4.506E+2	4.898E+3
	STD	**1.885**	1.873E+1	1.779E+1	8.716E+2	8.399E+1	6.404E+2	3.646E+2	9.752E+1	1.785E+1	1.445E+3
F18	AVG	**6.000E+2**	**6.000E+2**	**6.000E+2**	6.016E+2	**6.000E+2**	6.013E+2	6.010E+2	6.003E+2	**6.000E+2**	6.022E+2
	STD	2.080E-10	1.767E-5	**0**	2.226E-1	6.841E-2	2.271E-1	2.393E-1	1.930E-1	**0**	1.813E-1
F19	AVG	8.005E+2	8.009E+2	8.006E+2	8.047E+2	8.019E+2	8.040E+2	8.031E+2	8.005E+2	**8.004E+2**	8.061E+2
	STD	2.420E-1	3.619E-1	2.491E-1	4.190E-1	9.640E-1	5.771E-1	4.566E-1	1.618E-1	**1.164E-1**	8.147E-1
F20	AVG	**9.005E+2**	9.019E+2	9.010E+2	9.184E+2	9.027E+2	9.086E+2	9.064E+2	9.025E+2	9.013E+2	9.166E+2
	STD	7.771E-1	1.216	1.202	3.602	1.443	2.221	1.214	7.933E-1	**5.450E-1**	2.395
F21	AVG	6.981E+4	1.084E+5	7.071E+4	3.535E+9	1.369E+5	9.744E+8	7.874E+8	5.961E+4	**2.637E+4**	8.884E+9
	STD	2.069E+4	2.391E+4	2.077E+4	1.017E+9	2.163E+5	7.689E+8	3.135E+8	1.864E+4	**6.306E+3**	3.355E+9
F22	AVG	**2.028E+3**	2.143E+3	2.042E+3	5.725E+3	2.305E+3	3.906E+3	3.656E+3	2.164E+3	2.040E+3	8.997E+3
	STD	**6.148**	7.766E+1	1.394E+1	9.888E+2	2.438E+2	7.669E+2	4.943E+2	1.197E+2	1.679E+1	1.023E+3
F23	AVG	**2.234E+3**	2.811E+3	2.237E+3	1.633E+11	5.312E+3	1.548E+8	2.801E+7	2.834E+3	2.313E+3	4.824E+13
	STD	8.739	3.970E+2	**8.202**	3.540E+11	1.662E+3	5.275E+8	5.885E+7	5.508E+2	2.626E+2	6.548E+13
F24	AVG	2.636E+3	2.659E+3	2.637E+3	5.341E+3	2.699E+3	3.608E+3	3.563E+3	2.748E+3	**2.624E+3**	9.842E+3
	STD	**3.720E-1**	2.276E+1	1.124	6.743E+2	3.052E+1	4.989E+2	2.474E+2	8.639E+1	6.128E+1	2.720E+3
F25	AVG	**2.786E+3**	2.923E+3	3.471E+3	5.032E+3	5.679E+3	5.758E+3	3.060E+3	4.074E+3	2.788E+3	8.230E+3
	STD	**4.949E+1**	4.017E+2	7.226E+2	1.156E+3	1.670E+3	1.645E+3	1.312E+2	8.777E+2	5.572E+1	9.219E+2
F26	AVG	**2.600E+3**	2.602E+3	**2.600E+3**	8.269E+3	2.899E+3	5.653E+3	4.185E+3	2.755E+3	**2.600E+3**	2.260E+4
	STD	2.321E-4	4.765	4.811E-6	1.835E+3	5.563E+2	1.949E+3	5.814E+2	1.566E+2	**2.096E-12**	1.040E+4
F27	AVG	**2.953E+3**	2.957E+3	2.958E+3	3.712E+3	3.006E+3	3.219E+3	3.176E+3	3.045E+3	2.954E+3	4.172E+3
	STD	1.413E+1	**1.146E+1**	2.136E+1	1.375E+2	5.198E+1	1.454E+2	6.953E+1	5.868E+1	1.489E+1	4.647E+2

**Table 4:** Result of Wilcoxon rank-sum *p*-value and Friedman test.

		SAAO	AO	AOO	BKA	COA	DBO	PO	POA	TDO	TSO
CEC15	*p*-value	-	9.815E-4	1.053E-2	6.550E-4	6.550E-4	6.550E-4	6.550E-4	6.550E-4	4.073E-2	6.550E-4
	Friedman Value	**1.366667**	4.366667	2.166667	9.2	5.966667	7.8	7.2	4.8	2.6	9.533333
	Friedman Rank	**1**	4	2	9	6	8	7	5	3	10
CEC22	*p*-value	-	3.346E-3	3.815E-2	2.218E-3	3.346E-3	2.218E-3	2.218E-3	5.046E-2	4.768E-1	2.218E-3
	Friedman Value	**1.791667**	3.916667	2.916667	8.916667	5.666667	8.083333	6.75	4.958333	2.083333	9.916667
	Friedman Rank	**1**	4	3	9	6	8	7	5	2	10

**Table 5:** Results of SAAO and compared algorithms on engineering problem.

		SAAO	AO	AOO	BKA	COA	DBO	PO	POA	TDO	TSO
IBD	AVG	**1.745821E-4**	1.745823E-4	**1.745821E-4**	2.070608E-4	1.745841E-4	1.745825E-4	**1.745821E-4**	**1.745821E-4**	2.219362E-4	**1.745821E-4**
	STD	1.1930E-13	2.1018E-10	1.5525E-14	2.4087E-5	3.7722E-9	3.9329E-10	2.3531E-12	**0**	9.4475E-5	1.1930E-13
TCD	AVG	**30.149740**	30.217960	**30.149740**	30.748580	30.153270	30.217570	30.150280	**30.149740**	30.166820	**30.149740**
	STD	1.8059E-6	3.8863E-2	2.8410E-7	3.2926E-1	3.7326E-3	3.5788E-2	5.0898E-4	**0**	1.0030E-2	1.8059E-6
GTD	AVG	1.678856E-20	1.110937E-12	**1.312496E-24**	1.823367E-6	9.338011E-13	7.656260E-10	1.769581E-15	1.797288E-17	9.179766E-5	1.678856E-20
	STD	3.2980E-20	1.8457E-12	**4.3535E-24**	3.0569E-6	1.4461E-12	1.7669E-9	2.3903E-15	3.0264E-17	5.0280E-4	3.2980E-20

**Table 6:** Result of Ensemble-SAAO and baseline model.

Model	Accuracy	Precision	Recall	F1	AUC	LogLoss
Random Forest	0.9797	0.9255	0.8744	0.8992	0.9784	0.0795
Gradient Boosting	0.9791	0.9119	**0.8844**	0.8980	**0.9824**	0.0672
SVC	0.9797	0.9819	0.8191	0.8932	0.9656	0.0800
Extra Trees	0.9776	**0.9937**	0.7889	0.8796	0.9814	0.0889
AdaBoost	0.9771	0.9016	0.8744	0.8878	0.9813	0.6591
KNN	0.9453	0.8176	0.6080	0.6974	0.7987	1.1544
Decision Tree	0.9703	0.8660	0.8442	0.8550	0.9138	0.5697
Ensemble-SAAO	**0.9823**	0.9412	**0.8844**	**0.9119**	0.9805	**0.0645**

## Data Availability

The data obtained through the experiments are available upon request from the corresponding author.

## Nomenclature

AO—Aquila optimizer; AOO—animated oat optimization; SAAO—stagnation-aware Aquila optimizer; AEP—adaptive exploration probability; ISC—individual-level stagnation control; OBR—opposition-based reinitialization; OBL—opposition-based learning; N—population size; D (Dim)—problem dimensionality; t—current iteration index; T—maximum number of iterations; f(·)—objective (fitness) function; [LB, UB]—lower/upper search bounds; X—population matrix; X_i_—i-th candidate solution; X_best_—global best solution; X_M_—population mean used by AO; X_centroid_—population centroid used by the AOO-derived operators (numerically identical to X_M_); X_R_—randomly selected population member; X_new_, _i_—trial solution for agent i; c—cubic-decay control factor; G1, G2, QF—AO exploitation coefficients (Equations (12) and (13)); U = 0.00565 and ω = 0.005—fixed AO spiral constants; α = δ = 0.1–-AO exploitation constants; β = 1.5—Lévy exponent; Lévy(D)—D-dimensional Lévy flight step; A, B—sinusoidal/cosinusoidal oscillating bounds (AOO); m, L, e—per-agent seed mass, awn length, and eccentricity; R—rolling displacement; J—spring-ejection displacement; W—AOO dispersal perturbation vector; P_explore—adaptive exploration probability; τ_ISC = 30—individual-level stagnation threshold; τ_OBR = 100—severe-stagnation (reinitialization) threshold; noImprove[i]—per-agent non-improvement counter; ε = 10^−10^—global-improvement tolerance.
